# Contrasting parasite communities among allopatric colour morphs of the Lake Tanganyika cichlid *Tropheus*

**DOI:** 10.1186/1471-2148-13-41

**Published:** 2013-02-14

**Authors:** Joost AM Raeymaekers, Pascal I Hablützel, Arnout F Grégoir, Jolien Bamps, Anna K Roose, Maarten PM Vanhove, Maarten Van Steenberge, Antoine Pariselle, Tine Huyse, Jos Snoeks, Filip AM Volckaert

**Affiliations:** 1 Laboratory of Biodiversity and Evolutionary Genomics, University of Leuven, Ch. Deberiotstraat, 32, Leuven, B-3000, Belgium; 2 Zoological Institute, University of Basel, Vesalgasse 1, Basel, CH-4051, Switzerland; 3 Ichthyology Unit, Department of African Zoology, Royal Museum for Central Africa, Leuvensesteenweg 13, Tervuren, B-3080, Belgium; 4 ISE-M, UMR5554 CNRS, UR226 IRD, Université Montpellier II – CC 063, Montpellier Cedex 5, F-34095, France

**Keywords:** Adaptive divergence, Ectoparasite, Endoparasite, Ecological speciation, Host-parasite associations, Natural selection, Parasite-driven speciation, Sexual selection

## Abstract

**Background:**

Adaptation to different ecological environments is thought to drive ecological speciation. This phenomenon culminates in the radiations of cichlid fishes in the African Great Lakes. Multiple characteristic traits of cichlids, targeted by natural or sexual selection, are considered among the driving factors of these radiations. Parasites and pathogens have been suggested to initiate or accelerate speciation by triggering both natural and sexual selection. Three prerequisites for parasite-driven speciation can be inferred from ecological speciation theory. The first prerequisite is that different populations experience divergent infection levels. The second prerequisite is that these infection levels cause divergent selection and facilitate adaptive divergence. The third prerequisite is that parasite-driven adaptive divergence facilitates the evolution of reproductive isolation. Here we investigate the first and the second prerequisite in allopatric chromatically differentiated lineages of the rock-dwelling cichlid *Tropheus* spp. from southern Lake Tanganyika (Central Africa). Macroparasite communities were screened in eight populations belonging to five different colour morphs.

**Results:**

Parasite communities were mainly composed of acanthocephalans, nematodes, monogeneans, copepods, branchiurans, and digeneans. In two consecutive years (2011 and 2012), we observed significant variation across populations for infection with acanthocephalans, nematodes, monogeneans of the genera *Gyrodactylus* and *Cichlidogyrus*, and the copepod *Ergasilus* spp. Overall, parasite community composition differed significantly between populations of different colour morphs. Differences in parasite community composition were stable in time. The genetic structure of *Tropheus* populations was strong and showed a significant isolation-by-distance pattern, confirming that spatial isolation is limiting host dispersal. Correlations between parasite community composition and *Tropheus* genetic differentiation were not significant, suggesting that host dispersal does not influence parasite community diversification.

**Conclusions:**

Subject to alternating episodes of isolation and secondary contact because of lake level fluctuations, *Tropheus* colour morphs are believed to accumulate and maintain genetic differentiation through a combination of vicariance, philopatric behaviour and mate discrimination. Provided that the observed contrasts in parasitism facilitate adaptive divergence among populations in allopatry (which is the current situation), and promote the evolution of reproductive isolation during episodes of sympatry, parasites might facilitate speciation in this genus.

## Background

How organisms adapt to environmental conditions and how this process promotes speciation is a key question in evolutionary biology and speciation research. Ecologically based selection promotes adaptive divergence between populations, which may lead to the evolution of reproductive isolation and, ultimately, to ecological speciation [1-3]. Much of the evidence for ecological speciation comes from examples of adaptive radiation such as in three-spined stickleback, Darwin’s finches, Hawaiian honeycreepers, *Anolis* lizards, spiders on the Galapagos and Hawaiian archipelagos, and cichlid fishes [2,4-7]. The extreme diversification of cichlids, in particular the emergence of hundreds of species in the Great Lakes of the East African Rift Valley, has been puzzling biologists for decades. They comprise the most diverse species flocks of vertebrates on earth [8-10]. Their adaptive radiations have been attributed to the interaction of extrinsic factors such as ecological opportunities [6], lake-level fluctuations [11] and habitat diversity [12], as well as intrinsic factors in the form of adaptively relevant traits [13]. These include morphological, behavioural and physiological traits, and are targeted by natural or sexual selection.

Few relationships are as intimate as those between a parasite and its host, leading to strong ecological and evolutionary associations [14-17]. Parasites are increasingly recognized as important drivers of host diversity [18,19]. They also have been suggested to promote speciation [20-24] by triggering natural selection (by influencing host fecundity and host mortality [25-28]), as well as sexual selection (by influencing mate choice [28-30]). In vertebrates, both natural and sexual selection have been hypothesized to target the immune system [21,31]. The vertebrate immune system therefore classifies as a so-called ‘magic trait’ [32], which has the potential to initiate or accelerate speciation. Indeed, under the combined challenge of parasitism and associated mating decisions, the immune system strongly determines individual fitness. The result is that species diversification in vertebrates might have an important parasite-driven basis [21]. However, how often and how strong parasites are involved in speciation remains largely unknown [23].

Apart from studies in Lake Malawi [21,33] and Lake Victoria [25,26], and despite recent renewed interest in cichlid parasites [34-37], the influence of parasitism and the immune system on cichlid speciation has hardly been considered. One reason is that parasite-driven adaptation at the immunogenetic or behavioural level might be hard to detect. Nevertheless, most of the suggested drivers of cichlid radiations, such as habitat diversification (e.g. substrate type), trophic diversification (i.e. feeding strategies and diet) and social interactions (e.g. communication diversification, colour- and odour-based mate recognition) [38], are likely associated with shifts in parasite selection pressure. Indeed, diet [39-43], (social) behaviour [41,44], and environmental conditions [42,45] have proven to be important factors structuring fish parasite communities.

In this study, we investigate the possibility of a role for parasitism in the diversification among the highly fragmented eco-morphologically similar colour morphs (lineages) of *Tropheus* cichlids from Lake Tanganyika. Species of the genus *Tropheus* are obligate near-shore rock-dwelling philopatric fishes with low dispersal capacity [46-49]. Although currently six nominal species are recognized [50], the genus comprises more than 100 mostly allopatric colour morphs, which this taxonomical framework cannot unambiguously accommodate [51,52]. Therefore, and considering their stenotopy, populations are mostly referred to by their catch locality. The spatial distribution of the various *Tropheus* lineages shifted during lake level fluctuations in the Pleistocene [53], fragmenting populations during high stands, or forcing them into secondary contact during low stands [54,55]. These fluctuations strongly influenced the diversification of *Tropheus* populations [55,56]. Obviously, the same fluctuations might also affect communities of fish parasites. Fragmentation might induce parasite community diversification through local extinction driven by drift, or through species sorting and adaptation driven by local differences in biotic (e.g., the availability of intermediate and final hosts) or abiotic (e.g., turbidity, wave action and substrate type) conditions. As a result, fragmented *Tropheus* host populations likely end up with divergent parasite communities, experiencing differences in diversity or magnitude of infection. We hypothesize that, during episodes of isolation, these local parasite communities impose different selection pressures on their host populations, initiating or accelerating adaptive divergence. At subsequent phases of secondary contact between *Tropheus* populations, parasites might then facilitate host speciation by enhancing the evolution of reproductive isolation. Various mechanisms for parasite-driven speciation are possible [23], the likelihood of which depends on the composition of the merging parasite communities, as well as their effect on the fitness of parental types and hybrids.

**Figure 1:**
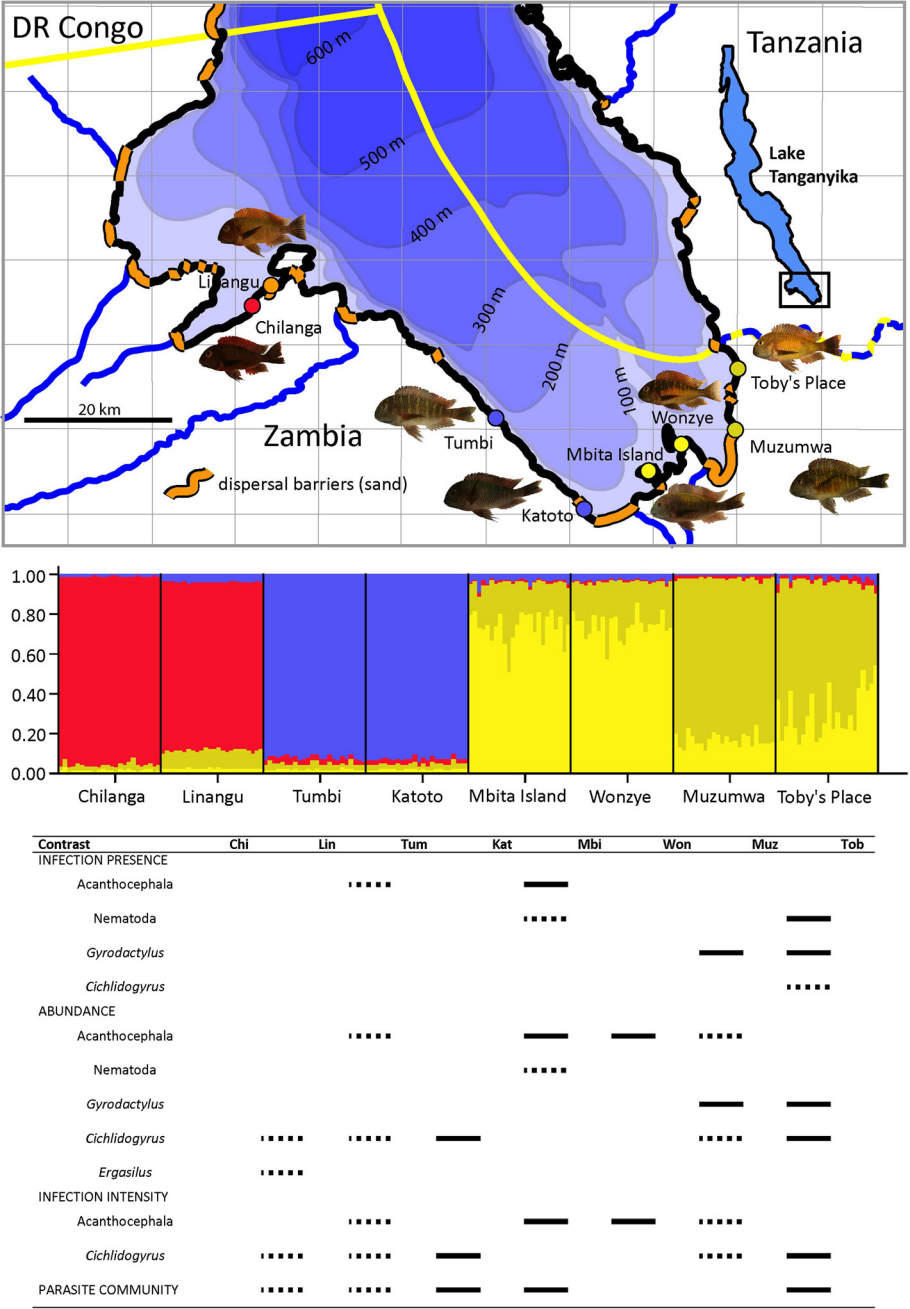
**A) Sites of eight *Tropheus *populations sampled along the Zambian shore of Lake Tanganyika in August-September 2011. **Black and sand-coloured shorelines indicate suitable rocky and unsuitable sand shores, respectively. Blue lines represent rivers, and the yellow line represents political borders. The six easternmost sites were resampled in August-September 2012. **B)** Bayesian analysis of the genetic structure of the eight populations. As previous genetic studies on these populations revealed that populations largely cluster according to colour morph [55,56,58,59], colour morph was used in the analysis as prior information. The colour of each of the four cluster corresponds to the predominant colour of the assigned individuals (i.e., red/orange for Chilanga and Linangu, blue for Tumbi and Katoto, light olive for Mbita Island and Wonzye Point, and dark olive for Muzumwa and Toby’s place). **C)** Visualization of significant differences (after correction for multiple testing) between neighbouring *Tropheus* populations for infection presence, abundance, infection intensity and parasite community composition. Full bars indicate that significant differences were observed in both sampling years. Dashed bars indicate that significant differences were only observed, or only investigated, in one sampling year.

We start our investigation by studying a first crucial prerequisite of parasite-driven speciation, i.e. that allopatric populations indeed experience different levels of infection. We also evaluate a second prerequisite, i.e. that these differences are stable in time, opening opportunities for consistent parasite-driven divergent selection and subsequent adaptive divergence. We do so by analyzing the spatial distribution of parasite taxa and the composition of parasite communities. For this purpose, we focussed on allopatric *Tropheus* colour morphs along the Zambian shore of Lake Tanganyika (Figure 1). These colour morphs could be regarded as potential founder populations of new species. This is reflected by their strong population genetic structure [48,49,55,57,58]. Eight populations, belonging to five different colour morphs, were screened in two consecutive years for metazoan ecto- and endoparasites in order to test for consistent parasite community diversification. Differences in parasite communities were compared with the degree of geographical isolation and the genetic structure of the *Tropheus* populations as assessed with microsatellite markers. We discuss the implications for the potential of parasite-driven speciation in cichlids. 

## Results

Parasites infecting the Zambian *Tropheus* populations were classified into nine categories (Tables 1 and 2). Parasites occurring at every single site included the gyrodactylid monogenean *Gyrodactylus* on skin and fins, the ancyrocephalid monogenean *Cichlidogyrus* and the copepod *Ergasilus* on the gills, and intestinal acanthocephalans and nematodes. Parasites which were not present at every single site included the monogenean *Urogyrus* in the urinary bladder, branchiurans in the gill cavity or on the opercula, some intestinal digeneans, and a number of unidentified helminthic cysts in skin, fin or gill tissue. While all categories were included in the multivariate analysis of parasite community composition, only the five categories occurring at every single site were analysed in more detail with a univariate analysis of infection patterns. 

**Table 1:** **Sampling site, substrate type, latitude, longitude, year, sample size and prevalence (%) for eight *Tropheus *populations sampled along the Zambian shore of Lake Tanganyika in August-September 2011 and 2012**

					***Endoparasites***				***Ectoparasites***				
**Site (substrate)**	**Latitude**	**Longitude**	**Year**	**N**_**T**_**/N**_**G**_	**Acanthocephala**	**Nematoda**	***Urogyrus***	**Digenea**	**cysts (Gills/Fins)**	***Gyrodactylus***	***Cichlidogyrus***	***Ergasilus***	**Branchiura**
Chilanga (r/s)	08° 33’ 22.4” S	30° 37’ 09.7” E	2011	50/40	92	16	0	0	5/4	2	97.5	57.5	0
Linangu (r/s)	08° 32’ 03.5” S	30° 38’ 25.2” E	2011	50/40	94	22	0	0	0/0	4	81.4	45	0
Tumbi (R/s)	08° 42’ 10.7” S	30° 55’ 20.9” E	2011	50/41	73.47	16.33	0	0	7.32/6	4	88.37	24.39	0
			2012	40/30	50	0	10	0	13.3/7.5	5	70	33.33	0
Katoto (R/ns)	08° 47’ 51.6” S	31° 01” 11.8” E	2011	55/40	30.77	13.46	7.27	1.92	0/1.82	5.45	100	47.5	2.5
			2012	40/31	72.50	25	7.50	2.50	0/2.5	12.50	93.55	29.03	0
Mbita Island (r/s)	08° 44’ 57.1” S	31° 05’ 14.2” E	2011	60/42	86.21	3.45	1.69	0	0/0	1.67	79.07	35.71	2.38
			2012	41/30	92.68	4.88	9.76	0	10/2.44	2.44	96.67	46.67	3.33
Wonzye Point (r-sr/ss)	08° 43’ 07.6” S	31° 08’ 12.6” E	2011	50/40	86	10	0	0	2.5/0	4	95.56	40	0
			2012	40/29	85	15	2.5	0	3.45/0	5	86.21	27.59	3.44
Muzumwa (r-sr/sss)	08° 42’ 05.7” S	31° 11’ 59.8” E	2011	50/45	95.91	10.20	2	0	0/4	20	95.56	31.11	0
			2012	40/30	92.5	5	2.5	0	6.67/0	20	90	36.67	0
Toby’s place (r/ss)	08° 37’ 18.9” S	31° 11’ 59.9” E	2011	50/40	90	6	0	0	0/2	4	76.19	25	2.5
			2012	40/30	77.5	5	5	0	0/0	2.50	66.67	30	3.44

Substrate type is categorized according to rock (r: small rocks; R: large rocks; sr: solid rock) and sediment (ns: no sediment; s: few sediment; ss: some sediment; sss: much sediment). N_T_: total number of individuals screened for parasites. N_G_: number of individuals screened for parasites on the gills. Groups of parasites which are known from Lake Tanganyika fishes [87], but which were not observed in this study, include pentastomids [88,89], cymothoid parasitic isopods, lernaeid copepods [90], cestodes [89,91] and leeches [92,93]. Of these, the isopods and copepods ( [90]; Vanhove pers. obs.), pentastomids [94], cestodes ( [95]; Vanhove & Pariselle, pers. obs.) were also observed in cichlids, as well as bivalves on the gills (Vanhove & Grégoir, pers. obs.) and the ancyrocephalid monogenean *Enterogyrus* sp. in the digestive tract (Pariselle, Vanhove, Bamps, Grégoir, Hablützel & Raeymaekers, unpublished data).

**Table 2:** **Mean abundance/median intensity in eight *Tropheus *populations sampled along the Zambian shore of Lake Tanganyika in August-September 2011 and 2012**

		**Endoparasites**				**Ectoparasites**					
**Site**	**Year**	**Acanthocephala**	**Nematoda**	***Urogyrus***	**Digenea**	**Gill cysts**	**Fin cysts**	***Gyrodactylus***	***Cichlidogyrus***	***Ergasilus***	**Branchiura**
Chilanga	2011	5.74 / 5	0.20 / 1	0.00 / -	0.00 / -	0.05 / 1	0.04 / 1	0.02 / 1	20.38 / 17	1.75 / 2	0.00 / -
Linangu	2011	6.54 / 5	0.26 / 1	0.00 / -	0.00 / -	0.00 / -	0.00 / -	0.08 / 2	3.44 / 4	0.60 / 1	0.00 / -
Tumbi	2011	1.57 / 2	0.22 / 1	0.00 / -	0.00 / -	0.10 / 1	0.06 / 1	0.12 / 3	6.81 / 5.5	0.46 / 1	0.00 / -
	2012	1.43 / 2	0.00 / -	0.13 / 1	0.00 / -	0.13 / 1	0.08 / 1	0.08 / 1.5	5.13 / 3	0.67 / 2	0.00 / -
Katoto	2011	0.48 / 1	0.17 / 1	0.07 / 1	0.02 / 1	0.00 / -	0.02 / 1	0.07 / 1	15.07 / 11	0.80 / 1	0.03 / 1
	2012	3.93 / 4	0.48 / 1	0.13 / 1	0.03 / 1	0.00 / -	0.03 / 1	0.25 / 2	14.06 / 10	0.48 / 1	0.00 / -
Mbita Island	2011	5.21 / 4	0.05 / 1.5	0.02 / 1	0.00 / -	0.00 / -	0.00 / -	0.02 / 1	5.44 / 6	0.83 / 1	0.05 / 2
	2012	10.95 / 9.5	0.05 / 1	0.22 / 2	0.00 / -	0.1 / 1	0.02 / 1	0.05 / 2	15.8 / 11	1.17 / 2	0.03 / 1
Wonzye	2011	3.48 / 4	0.10 / 1	0.00 / -	0.00 / -	0.03 / 1	0.00 / -	0.08 / 2	6.8 / 6	0.63 / 1	0.00 / -
	2012	4.88 / 5	0.15 / 1	0.05 / 2	0.00 / -	0.03 / 1	0.00 / -	0.08 / 1.5	6.55 / 5	0.41 / 1	0.03 / 1
Muzumwa	2011	6.67 / 4	0.12 / 1	0.02 / 1	0.00 / -	0.00 / -	0.06 / 1.5	0.38 / 1	14.47 / 10	0.53 / 1	0.00 / -
	2012	6.15 / 5	0.05 / 1	0.03 / 1	0.00 / -	0.17 / 2.5	0.00 / -	0.48 / 2	12.57 / 11	0.5 / 1	0.00 / -
Toby’s place	2011	6.62 / 5	0.06 / 1	0.00 / -	0.00 / -	0.00 / -	0.02 / 1	0.08 / 2	2.98 / 3	0.40 / 1.5	0.03 / 1
	2012	6.38 /7	0.05 / 1	0.13 / 2.5	0.00 / -	0.00 / -	0.00 / -	0.03 / 1	4.17 / 4	0.43 / 1	0.03 / 1

### Infection patterns

**Figure 2:**
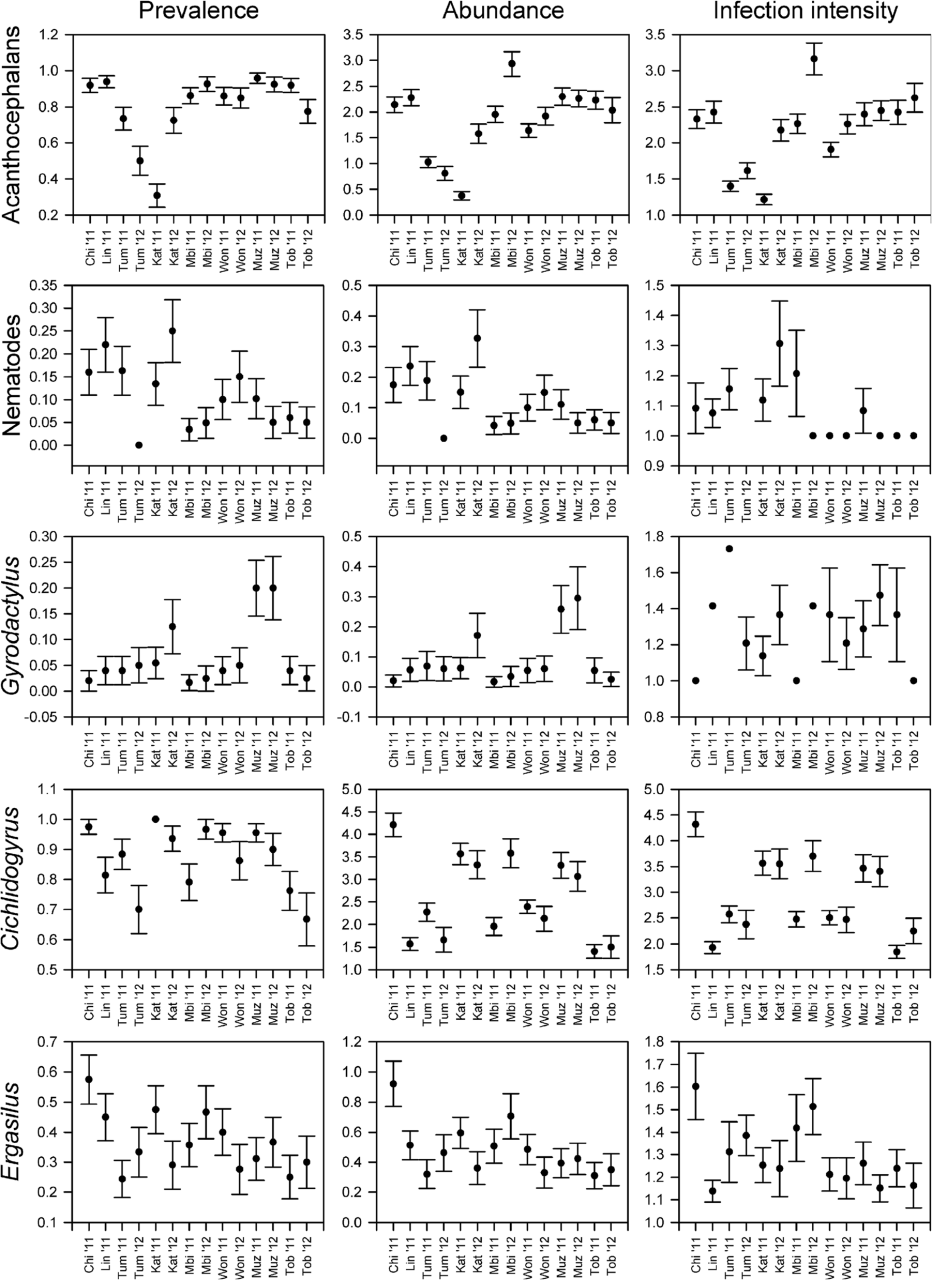
**Prevalence (left), mean abundance (middle; square-root transformed) and mean infection intensity (right; square-root transformed) of acanthocephalans, nematodes, *Gyrodactylus*, *Cichlidogyrus*, and *Ergasilus *in eight *Tropheus *populations from southern Lake Tanganyika. **Error bars represent standard errors.

Controlling for standard fish length, sex, day of dissection, sampling year and observer, significant differences in infection parameters between sites were detected for the five major parasite categories (Table 3). For acanthocephalans, the differences were due to lower infection presence (prevalence), abundance and infection intensity at Tumbi and Katoto (i.e., the blue morph) compared to all other sites (Figure 2). For nematodes, infection presence and abundance were significantly higher at Chilanga, Linangu and Katoto (i.e., some of the western colour morphs) as compared to Toby’s place, Muzumwa and Mbita Island (i.e., the eastern colour morphs). For *Gyrodactylus*, infection presence and abundance were significantly higher at Muzumwa than elsewhere (Figure 2). For *Cichlidogyrus*, there were various significant contrasts without an obvious association with colour variation or geography. For *Ergasilus*, abundance was higher at Chilanga (i.e., the red morph) than at most other sites (Figure 2). Finally, an analysis of differences between neighbouring sites revealed that all pairs of neighbours (regardless of colour morph) differed significantly in infection parameters for at least one group of parasites (Figure 1). 

**Table 3:** **Fixed effects of general and generalized linear models for infection levels in eight *Tropheus *populations**

				**Infection presence**		**Abundance**		**Infection intensity**	
**Parasite group**	**Effect**	**Num DF**	**Den DF**	**F**	**P**	**F**	**P**	**F**	**P**
Acanthocephalans	site	7	605/483	9.01	**< 0.0001**	23.46	**< 0.0001**	11.92	**< 0.0001**
	day	2	605/483	0.76	0.4673	1.02	0.3622	01.25	0.2886
	sex	1	605/483	0.01	0.9100	0.04	0.8484	0.04	0.8351
	SL	1	605/483	12.62	**0.0004**	57.06	**< 0.0001**	47.92	**< 0.0001**
	year(site)	6	605/483	5.02	**< 0.0001**	7.57	**< 0.0001**	5.02	**< 0.0001**
Nematodes	site	7	605/47	2.38	**0.0210**	2.64	**0.0106**	1.32	0.2640
	day	2	605/47	0.63	0.5336	0.78	0.4591	2.41	0.1005
	sex	1	605/47	1.09	0.2974	1.36	0.2434	1.17	0.2843
	SL	1	605/47	0.10	0.7557	0.05	0.8185	1.39	0.2436
	year(site)	6	605/47	0.89	0.5011	2.19	**0.0428**	0.74	0.6009
*Gyrodactylus*	site	7	616/24	3.95	**0.0003**	5.47	**< 0.0001**	0.83	0.5692
	day	2	616/24	0.90	0.4091	0.37	0.6927	4.88	**0.0167**
	sex	1	616/24	0.30	0.5811	0.09	0.7607	1.16	0.2926
	SL	1	616/24	0.26	0.6093	0.27	0.6016	1.25	0.2751
.	year(site)	6	616/24	0.35	0.9071	0.60	0.7344	0.93	0.4933
*Cichlidogyrus*	site	7	476/418	2.85	**0.0064**	18.85	**< 0.0001**	15.14	**< 0.0001**
	day	2	476/418	2.80	0.0617	5.50	**0.0043**	2.54	0.0803
	sex	1	476/418	3.62	0.0578	7.58	**0.0061**	4.00	**0.0462**
	SL	1	476/418	6.60	**0.0105**	77.90	**< 0.0001**	74.38	**< 0.0001**
	year(site)	6	476/418	2.80	**0.0108**	3.68	**0.0014**	1.73	0.1131
*Ergasilus*	site	7	476/158	1.73	0.1001	2.40	**0.0201**	1.86	0.0792
	day	2	476/158	0.70	0.4984	0.14	0.8729	1.36	0.2602
	sex	1	476/158	0.15	0.6978	0.10	0.7479	0.00	0.9537
	SL	1	476/158	0.58	0.4485	3.28	0.0708	5.30	**0.0227**
	year(site)	6	476/158	0.70	0.6489	0.55	0.7696	0.23	0.9667

Fixed effects included sampling site, processing day, sex, standard length (SL), and sampling year (nested in site). Observer effects were included as random (not shown), except for *Gyrodactylus* sp. for which there was only one observer. The model for infection presence assumes a binomial distribution and models the logit of the probability of infection. For the models for abundance and infection intensity, the dependent variable was square-root transformed. Note that the denominator degrees of freedom (Den DF) are higher for the infection presence and abundance model (before the dash) than for the infection intensity model (after the dash). P-values in bold indicate significance at α = 0.05.

Next to the effect of site, the analyses revealed a few other significant relationships (Table 3). Infection parameters increased with the standard length of the fish for acanthocephalans and *Cichlidogyrus* (infection presence, abundance and intensity), and for *Ergasilus* (intensity). Day of dissection affected the numbers of *Gyrodactylus* (larger infection intensities on the second day than on the first day). Males harboured more *Cichlidogyrus* individuals than females (abundance and infection intensity). Finally, significant year-to-year variation in infection parameters within sites was detected for acanthocephalans, nematodes and *Cichlidogyrus*. However, this variation was generally smaller than the variation observed between sites (Table 3; Figure 2), and differences between neighbouring sites were often stable over time (Figure 1).

### *Population genetics of* Tropheus *spp*

**Figure 3:**
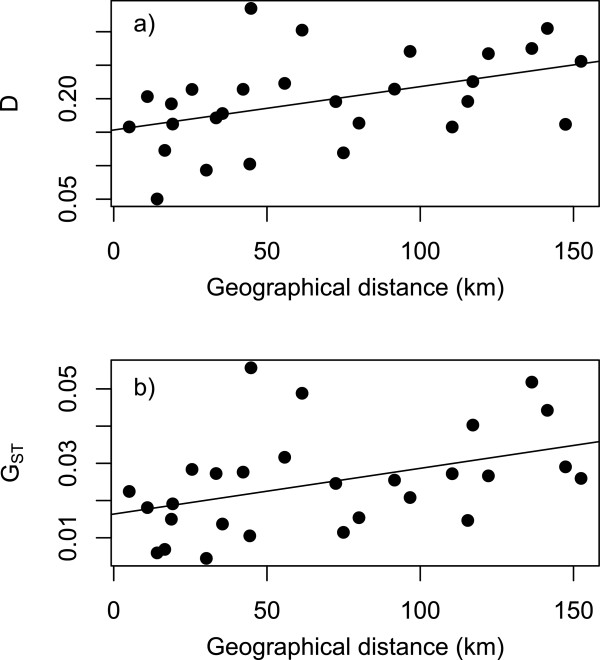
**Isolation-by-distance in eight *Tropheus *populations from southern Lake Tanganyika. A) **Geographical distance along the shoreline vs. genetic differentiation as quantified with pairwise D; **B)** Geographical distance along the shoreline vs. standardized variance in allele frequencies (G_ST_).

Seven *Tropheus* populations harboured comparable levels of genetic diversity (allelic richness: 11.9-14; observed heterozygosity: 0.78-0.83), while the population of Muzumwa had slightly lower values (allelic richness: 9.7; observed heterozygosity: 0.73). Analysis of genetic structure revealed significant global values for G_ST_ and D (G_ST_ = 0.042, P < 0.001, 95% CI = [0.034-0.051]; D= 0.22, P < 0.001, 95% CI = [0.19-0.24]). All pairwise G_ST_ and D values (28 pairs) were also significant (all P < 0.001) and revealed a significant isolation-by-distance pattern (G_ST_ : R = 0.43, P = 0.022; D: R = 0.45, P = 0.009; Figure 3). 

Bayesian clustering analyses (Figure 1) showed an optimal ln likelihood value for K=4. In the West, the Chilanga population clustered with Linangu (i.e., the red and the orange morph), while Tumbi clustered with Katoto (i.e., the blue morph). In the East, Mbita Island and Wonzye Point (i.e., the light-olive morph) formed a cluster, while Muzumwa clustered with Toby’s place (i.e., the dark-olive morph).

### Parasite community composition

A global analysis of relative differences in parasite community composition based on pairwise Hellinger distances revealed strong differences between host populations for both sampling years (Table 4). This analysis also revealed differences between all pairs of neighbouring populations, except between Mbita Island and Wonzye Point (both belonging to the light-olive morph), and between Wonzye Point and the dark-olive population from Muzumwa (Figure 1). 

**Table 4:** **Permutational multivariate analysis of variance on Hellinger distances between parasite communities in individuals from eight (2011) or six (2012) *Tropheus *populations**

**Year**	**Effect**	**Num DF**	**Den DF**	**SS**	**MS**	**F**	**R**^**2**^	**P**
2011	site	7	307	16.84	2.41	12.22	0.21	**<0.0001**
	day	2	307	0.81	0.40	2.05	0.01	0.08
	sex	1	307	0.23	0.23	1.18	0.003	0.28
	SL	1	307	0.40	0.40	2.04	0.005	0.11
2012	site	5	168	4.42	0.88	3.05	0.08	**0.0003**
	day	1	168	0.05	0.05	0.17	0.001	0.97
	sex	1	168	0.65	0.65	2.24	0.01	0.07
	SL	1	168	0.29	0.29	0.99	0.005	0.39

The model included sampling site, processing day, sex, and standard length (SL). P-values in bold indicate significance at α = 0.05.

**Figure 4:**
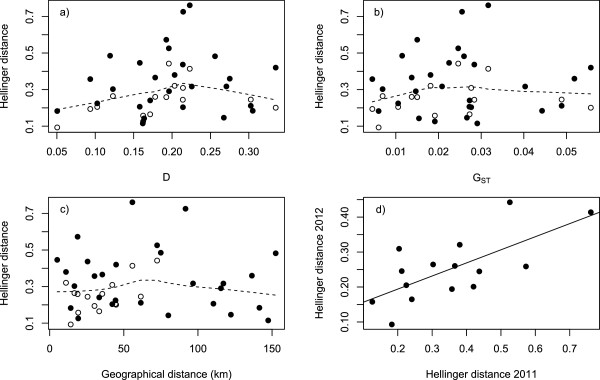
**Determinants of parasite community differentiation (Hellinger distance) among eight *Tropheus *populations from southern Lake Tanganyika. A)** Genetic differentiation as quantified with pairwise D vs. Hellinger distance; **B)** Standardized variance in allele frequencies (G_ST_) vs. Hellinger distance; **C)** Geographical distance along the shoreline vs. Hellinger distance; **D)** Hellinger distance as quantified in 2011 vs. Hellinger distance as quantified in 2012. Panels A, B and C combine data from 2011 (black dots) with data from 2012 (white dots). Dashed lines (superimposed on non-significant relationships) were obtained with a lowess function. Full lines (superimposed on significant relationships) represent least-square linear regression lines.

Correlations between parasite community composition and the genetic structure of host populations were positive, but non-significant (Table 5; Figure 4). The correlation between parasite community composition and geographical distance along the shoreline was non-significant in 2011 (i.e., including all populations), and significantly positive in 2012 (i.e., only including the six easternmost populations). Hellinger distances in 2011 were positively correlated with Hellinger distances in 2012 (R = 0.69, P = 0.0261; Figure 4), indicating that parasite communities were stable over this period of time. Accordingly, pairwise Hellinger distances between parasite communities from the same site but different years were on average smaller than distances between neighbouring sites, and smaller than distances between all other site pairs (Mantel test between all distances and a vector assigning values 0,1 and 2 to each of these respective categories: R = 0.20; P = 0.028). 

**Table 5:** **Mantel correlations between parasite community differentiation (Hellinger distance), the genetic structure of *Tropheus *host populations (pairwise D and pairwise G**_**ST**_**), and geographical distance along the shoreline**

	**Hellinger distance (2011)**		**Hellinger distance (2012)**	
	**R**	**P**	**R**	**P**
D	0.13	0.2803	0.37	0.1396
G_ST_	0.02	0.4255	0.20	0.2589
Geographical distance	−0.14	0.6983	0.55	**0.0369**

Significant P-values are in bold.

## Discussion

There are three main prerequisites for parasite-driven speciation [1-3,23]. First, different populations or ecotypes should experience divergent infection levels. Second, divergent infection levels should cause divergent selection and facilitate adaptive divergence. Third, parasite-driven adaptive divergence should facilitate the evolution of reproductive isolation. Below we discuss indications for parasite-driven speciation in *Tropheus* and other cichlid species by providing an overview of the support currently available for each of these prerequisites.

### First prerequisite

Divergent parasite communities have been described in closely related sympatric cichlid fish from Lake Malawi [21] and Lake Victoria [25]. The differences in these systems were mainly caused by intestinal nematodes, and by gill parasites such as ergasilid copepods and the monogenean *Cichlidogyrus*. Our analyses of infection levels and parasite community composition revealed considerable variation in parasitism among eight *Tropheus* populations from Lake Tanganyika, belonging to five allopatric colour morphs. Two common groups of endoparasites (acanthocephalans and nematodes) and three common ectoparasites (the monogeneans *Gyrodactylus* and *Cichlidogyrus* and the copepod *Ergasilus*) contributed to this effect. A number of less common parasite groups with a patchy distribution (e.g. Digenea only observed at Katoto) further enhanced the differences between sites. The differences remained after correction for observer, hence representing a true biological effect. Most of the differences also remained after correction for sex and standard length, which implies that they were not due to the size distribution or sex bias of the sampled populations. As all samples were collected within three weeks, and nearby sites were often visited in the same week or on the same day, it is unlikely that seasonal environmental fluctuations represented a main contributor to the observed variation. We conclude that the allopatric *Tropheus* populations showed true parasitological differences, supporting the first prerequisite for parasite-driven speciation. This is a conservative conclusion, because the differences between host populations in parasite species composition are likely to increase with higher taxonomic resolution. Preliminary data on *Cichlidogyrus* do suggest this, as the *Tropheus* populations concerned harbour at least seven *Cichlidogyrus* species, most of which occur at different localities (Grégoir et al., unpublished).

### Second prerequisite

Divergent infection levels open opportunities for parasite-driven divergent selection and subsequent adaptive divergence, i.e. the second prerequisite for parasite-driven speciation. Importantly, only consistent parasite-mediated selection might lead to adaptive divergence [23]. This requires a reasonable degree of temporal stability of the parasite metacommunity. Our analysis over a one year time span showed no major shifts in parasite distributions, and hence was indicative for temporal stability. The stability of communities of fish parasites is influenced by the environment, which for lake cichlid systems might include factors such as the availability of host species, substrate type, turbidity and temperature. Our study sites certainly differ in these respects, some environmental characteristics of which are stable. Substrate type (Table 1), for instance, is a stable factor, and for *Tropheus* as an algae scraping cichlid, the variation from pebbles to plain rock may highly influence infection risk. At the same time substrate type also influences hiding opportunities and predation risk, and hence it can strongly influence the chances of parasites to pass from intermediate hosts (e.g. cichlids) to final hosts (e.g. fish-eating birds). Furthermore, the stability of the parasite metacommunity might also depend on host dispersal. In confirmation of previous studies [55,56,58,59], our genetic analysis showed that host dispersal was limited, especially between populations from different colour morphs. Furthermore, we observed a non-significant correlation between parasite community diversification and the genetic structure of host populations, suggesting that the relationship between parasite distributions and host dispersal is weak. This implies that host dispersal might be too low to homogenize parasite communities, or that the local environment is a stronger determinant of parasites distributions than host dispersal. Either way, it is likely that at least part of the parasite community is restricted to local *Tropheus* populations, imposing divergent selection.

Reversing the arrow of causality, we might as well consider the possibility that contrasting parasite communities *reduce* host dispersal. However, as spatial isolation represents a much stronger factor reducing host dispersal, this relationship cannot be unambiguously analysed. It also remains unclear whether divergent infection levels can facilitate chromatic differentiation, as seems to be the case in a pair of cichlid species from Lake Victoria [25]. Interestingly, neighbouring populations belonging to different colour morphs had highly divergent parasite communities (Figure 1), but neighbouring populations belonging to the same colour morph were also rather divergent in parasitism (with the exception of the light-olive populations - i.e. the only pair without a major barrier to *Tropheus* dispersal; Figure 1). Therefore it might be that parasites represent a stronger diversifying force than the factors underlying chromatic differentiation. Alternatively, parasites themselves might be more influenced by spatial isolation or environmental heterogeneity than the factors underlying chromatic differentiation.

The potential to *adapt* to parasite-mediated selection might include adaptation at the behavioural as well as the immunological level. A number of studies provide evidence for the potential of adaptation to contrasting parasite environments in other teleosts, in particular through specialized immune functions (reviewed in [23,29]. In cichlids from Lake Malawi, it was shown that two sympatric species harbouring divergent parasite communities were different at the immunogenetic level [21]. So far immunogenetic adaptation has not been investigated in *Tropheus*.

### Third prerequisite

In general, reproductive isolation might be composed of one or multiple reproductive barriers, including geographical isolation, habitat choice, assortative mate choice, and natural selection against migrants or hybrids [1,3,60,61]. Mechanisms of how parasites might facilitate host speciation include reduced viability or fecundity of immigrants and hybrids, assortative mating as a pleiotropic by-product of immunogenetic adaptation, and ecologically-based sexual selection [23].

At the moment, there is no evidence for the evolution of parasite-mediated reproductive isolation in *Tropheus*. A combination of allopatry, philopatric and stenotopic behaviour and mate discrimination are believed to maintain the differentiation between the colour morphs [59,62]. Partial colour-assortative female mate choice has been observed for the populations in our study area based on paternity tests in a human-mediated admixis of colour morphs in the harbour of Mpulungu [59,63], as well as in mating trials among colour morphs from Moliro, Chimba, Chaitika, Nakaku and Mbita Island [62,64]. However, the females in these studies could also rely on other cues, in particular olfaction and sound which have been proposed to influence mate choice in cichlids as well [65,66]. As such there is no direct evidence that colour influences mating decisions in *Tropheus*. Currently, there is also no indication that colour *intensity* affects intra-population mating decisions in *Tropheus*[67]. Therefore additional, nonexclusive mechanisms affecting mating decisions might be considered, including those which invoke a role for parasitism. Assortative odour-based mate choice linked to the immune competences of potential mates [20] represents one pathway of how reproductive isolation might evolve among populations with divergent parasite communities. A study on a pair of Lake Victoria cichlids suggested that parasite-mediated sexual selection might contribute to the divergence of female mating preferences for male coloration, strengthening reproductive isolation [25]. Consistent with parasite-mediated sexual selection, males had higher parasite loads (e.g. *Cichlidogyrus*) than females in this system [26], something which we also observed in this study.

The evolution of parasite-mediated reproductive isolation also depends on the fitness of parental types and hybrids [23]. There is empirical evidence that hybrids differ from purebreds in parasite infection rates by being more or intermediately susceptible [68-71], or by being more resistant [72,73] to a particular parasite species. Furthermore, hybrid genotypes within a population can differ from each other in parasite susceptibility [68]. In *Tropheus*, various scenarios with contrasting outcome may be observed, ranging from full reproductive isolation between colour morphs when parental types are able to deal better with parasites than hybrids, to considerable levels of introgression and even hybrid speciation when hybrids are able to deal better with parasites than parental types. The occurrence of at least two ancient *Tropheus* hybrid zones within our study area is interesting for further investigation in this context [56].

## Conclusions

Allopatric *Tropheus* populations revealed considerable and consistent variation in parasite community composition. The observation of divergent parasite communities between distinct colour morphs suggests that *Tropheus* represents a good system for parasite-mediated adaptive divergence and speciation. At the moment it is unknown whether the current diversification in *Tropheus* has been influenced by differential parasite load in the past. Neither do we know whether the current differences in parasitism contribute to adaptive divergence and speciation in the future. *Tropheus* colour morphs have been subject to alternating episodes of isolation and secondary contact because of lake level fluctuations. Our results imply that during phases of secondary contact, merging populations might come in contact with different parasite communities. The outcome of this process is hard to predict, but it will likely affect the fitness of different colour morphs and their potential hybrids differentially. This certainly influences the process of admixing; further studies are needed to evaluate how this might influence the evolution of reproductive isolation. Our future work will aim at increasing the taxonomic resolution of parasite identification, improving our understanding of the factors structuring parasite communities, and evaluating the likelihood of immunogenetic adaptation.

## Methods

### Sampling

Sampling was conducted during August-September 2011 and 2012 along the Zambian shoreline of Lake Tanganyika (Table 1, Figure 1). In 2011, eight sites were included, while in 2012 the six easternmost sites were re-sampled. The choice of the eight sites was based on the distribution of five allopatric *Tropheus* colour morphs [46,58,59,62,64]: the red morph (sampled at Chilanga), the orange ‘Llangi’-like morph (sampled at Linangu), the blue morph (sampled at Tumbi and just west of Katoto), the light-olive or yellow-blotched morph (sampled at Mbita Island and Wonzye Point), and the dark-olive morph (sampled at Muzumwa and Toby’s place). The morphs are to variable degrees isolated by distance and habitat unsuitable for *Tropheus* (Figure 1). Nuclear and mitochondrial phylogenies reveal partially independent evolutionary lineages for these allopatric colour morphs [55,56,59]. Fifty to sixty fish per site were caught by chasing fish into standing nets. After transport in oxygenated water to a near-shore tank facility (Toby’s place), the fish were kept in tanks of 0.8 m × 0.8 m × 1.2 m depth or 2.0 m × 0.8 m × 1.2 m depth. Before usage, tanks were cleaned, dried and filled with lake water. At every site, substrate type was determined according to rock type (small, large or solid rock) and sediment presence (no, few, some or much sediment).

### Parasitological survey

Within three days after capture, all live stock *Tropheus* were dissected. The parasitological survey was performed in the field using a field stereomicroscope. Individual fish were killed with an overdose of MS222. For each fish, the observers performing the dissections were recorded in order to keep track of observer bias. The outer surface of the fish was screened by a single observer (JAMR). The gills were screened by two observers in 2011 and four observers in 2012. The intestines were screened by four observers in 2011 and four observers in 2012. The day of dissection after capture (day 0, 1 or 2) was recorded in order to keep track of changes in parasitological parameters while the fish were kept in the tanks. The dissection of each fish started with screening its outer surface for monogeneans and crustaceans (copepods, branchiurans, isopods), and any kind of helminthic cyst. The mouth cavity was then inspected for parasitic isopods and branchiurans. At least fourty fish in 2011 and thirty fish in 2012 per site were inspected for gill parasites including branchiurans, copepods, bivalves, monogeneans, and any kind of helminthic cyst. To do so, the gills were immediately dissected and stored on 100% ethanol for later processing. At least fifty fish in 2011 and fourty fish in 2012 per site were screened for intestinal monogeneans, digeneans, acanthocephalans, nematodes, and any kind of helminthic cysts. To do so, stomach, intestines, gall and urinary bladder were immediately dissected and inspected in a petridish with lake water. Finally, the sex of the fish was determined by visual inspection of the genital papilla and gonad development. Processed fish were wrapped in cheese cloth, preserved on formalin, and deposited in the RMCA as vouchers (samples 2011: collection RMAC B1.23; samples 2012: collection RMAC B2.38).

All parasites were counted and identified to genus or class level and preserved as follows. Monogeneans were isolated using dissection needles and were either mounted on slides in ammonium picrate glycerine [74] for further morphological research, or stored on 100% analytical ethanol (EtOH). Acanthocephalans and nematodes were stored on 80% EtOH, while intestinal monogeneans, branchiurans, copepods, any kind of helminthic cysts, bivalves and unknown groups were stored on 100% EtOH.

### Population genetics

Genomic DNA of 24 individuals per population (all captured in 2011) was isolated from 10–20 mg fin tissue with the Nucleospin Tissue kit (Macherey & Nagel, Düren, Germany) following the manufacturer’s recommendations. Ten neutral microsatellite loci were amplified in three multiplex reactions with annealing temperatures 54°C (reaction I and III) or 56°C (reaction II) using the QIAGEN PCR kit. Reaction I amplified loci Ppun5 and Ppun7 [75], and locus Pzeb3 [76]. Reaction II amplified loci HchiST06, HchiST38, HchiST68 and HchiST94 [75]. Reaction III amplified loci TmoM11 [77], UME003 [78], and UNH130 [79]. Genotyping was performed using an ABI 3130 Sequencer (Applied Biosystems). Allele sizes were estimated using Genemapper v4.0 (Applied biosystems) and verified visually.

Allelic richness (AR) and expected (H_E_) and observed (H_O_) heterozygosity were calculated for all loci and sampling sites using Arlequin v3.5 [80]. Tests for linkage disequilibrium among all pairs of loci were performed using the Markov Chain algorithm implemented in Genepop v4.0, with 10^4^ dememorizations, 500 batches and 5 000 iterations per batch. To test for significant deviations from Hardy-Weinberg equilibrium, the exact test implemented in Arlequin was used, with 10^6^ steps in the Markov Chain and 10^4^ dememorizations per population.

Global and pairwise population differentiation (D; [81]) and the global and pairwise standardized variance in allele frequencies (G_ST_) were quantified using the R package DEMEtics [82]. Confidence intervals for all estimates were obtained by bootstrapping over loci. Furthermore, a structure analysis in Structure v2.3 [83] was run in order to determine the most likely number of differentiated clusters (1 <*K* < 8). The analysis used an admixture model with correlated allele frequencies. Colour morph was used as prior information, given the suboptimal resolution of microsatellite markers, and given that the populations in this study have been repeatedly shown to cluster genetically according to colour morph based on mtDNA (i.e., control region [55,56,59]), AFLP [56,58], or microsatellite data [55,59]. For every *K*, five replicates with 10^6^ iterations after a burnin of 10^5^ iterations were run. The optimal *K* was determined using Bayes’ Rule.

### Data analysis

Prevalence, infection presence, abundance and infection intensity were calculated for each group of parasites following the terminology of Rózsa et al. 2000 [84]. Infection presence (yes/no) was analyzed with a generalized linear model assuming a binomial error distribution using proc GLIMMIX in SAS v9.1 (SAS Institute, Cary, NC, USA). Site, sex, day of dissection and sampling year were included in the model as fixed factors, and observer as a random block factor. Sampling year was nested in site in order to test for local year-to-year variation in infection presence. The standard length of the fish was added to the model as a covariate. In case of overall significance of the site effect, *post hoc* comparisons of least-square infectivity means between pairs of sites were computed. Abundance and infection intensity were square-root transformed in order to improve normality, and compared between sites using a general linear model using proc MIXED in SAS. As above, site, sex, day of dissection and sampling year (nested in site) were included as fixed factors, observer as a random block factor, and standard length as a covariate. In case of overall significance of the model, *post hoc* comparisons of mean ranks for all pairs of sites were computed.

Dissimilarities in parasite community composition between host individuals were assessed by calculating Hellinger distances using the R library vegan [85]. Hellinger distances are based on square-rooted proportional abundances [80], and therefore reflect relative differences in parasite community composition. For each year, a permutational multivariate analysis of variance on Hellinger distances with factors host population, sex, day of dissection and size as a covariate was performed using the Adonis function in vegan [86]. Statistical significance was obtained through 10^4^ permutations of the data. This analysis was then repeated for each pair of host populations separately, applying a significance level corrected for multiple comparisons (2011: 28 pairwise comparisons, α = 0.0018; 2012: 15 comparisons, α = 0.0033). In order to test the expectation that the differences in parasite communities are correlated with geographical isolation and the extent of genetic differentiation between host populations, pairwise Hellinger distances between parasite communities were compared with pairwise D, pairwise G_ST_ and pairwise geographical distances between the host populations. In order to test for the stability of parasite communities over time, pairwise Hellinger distances between parasite communities for 2011 were correlated with the distances for 2012. All correlations were tested for significance using a Mantel test implemented in vegan.

Because *Tropheus* populations from neighbouring sites might merge as a result of lake level fluctuations, differences in parasite distributions might crucially affect the evolution of parasite-driven reproductive barriers. Therefore all significant differences in infection presence, abundance, infection intensity and parasite community between the seven pairs of neighbouring sites were visualized in a single figure, along with their degree of geographical isolation (i.e. length of unsuitable stretches of sand, or length of suitable stretches of rocky outcrops). Habitat suitability was determined based on observations on site complemented with visual inspection of satellite pictures.

## Competing interests

The authors declare that they have no competing interests.

## Authors’ contributions

JAMR and PH coordinated and designed the study. JAMR, PH, AFG, JB and AKR performed the sampling and dissections at Lake Tanganyika. JAMR, PH, AFG, JB, AKR, MPMV, MVS, AP and TH identified and counted the parasites. JAMR, PH, and AFG analyzed the data. JAMR, PH, AFG, MPMV, MVS, AP, TH, JS and FAMV participated in the coordination of the study and wrote the manuscript. All authors read and approved the final manuscript.

## References

[B1] Hendry AP Ecological speciation! Or the lack thereof? Can J Fish Aquat Sci 2009 66 8 1383 1398

[B2] Schluter D The Ecology of Adaptive Radiation 2000 Oxford: Oxford University Press

[B3] Rundle HD Nosil P Ecological speciation Ecol Lett 2005 8 3 336 352

[B4] Gillespie R Community assembly through adaptive radiation in Hawaiian spiders Science 2004 303 5656 356 359 14726588 10.1126/science.1091875

[B5] De Busschere C Hendrickx F Van Belleghem SM Backeljau T Lens L Baert L Parallel habitat specialization within the wolf spider genus Hogna from the Galapagos Mol Ecol 2010 19 18 4029 4045 20695996 10.1111/j.1365-294X.2010.04758.x

[B6] Seehausen O African cichlid fish: a model system in adaptive radiation research Proc R Soc B-Biol Sci 2006 273 1597 1987 1998 10.1098/rspb.2006.3539 PMC1635482 16846905

[B7] Losos JB Jackman TR Larson A de Queiroz K Rodríguez-Schettino L Contingency and determinism in replicated adaptive radiations of island lizards Science 1998 279 5359 2115 2118 9516114 10.1126/science.279.5359.2115

[B8] Koblmüller S Schliewen UK Duftner N Sefc KM Katongo C Sturmbauer C Age and spread of the haplochromine cichlid fishes in Africa Mol Phylogenet Evol 2008 49 1 153 169 18582582 10.1016/j.ympev.2008.05.045

[B9] Turner GF Seehausen O Knight ME Allender CJ Robinson RL How many species of cichlid fishes are there in African lakes? Mol Ecol 2001 10 3 793 806 11298988 10.1046/j.1365-294x.2001.01200.x

[B10] Kornfield I Smith PF African cichlid fishes: Model systems for evolutionary biology Annu Rev Ecol Syst 2000 31 163-+

[B11] Sturmbauer C Baric S Salzburger W Ruber L Verheyen E Lake level fluctuations synchronize genetic divergences of cichlid fishes in african lakes Mol Biol Evol 2001 18 2 144 154 11158373 10.1093/oxfordjournals.molbev.a003788

[B12] Kocher TD Adaptive evolution and explosive speciation: the cichlid fish model Nat Rev Genet 2004 5 4 288 298 15131652 10.1038/nrg1316

[B13] Salzburger W The interaction of sexually and naturally selected traits in the adaptive radiations of cichlid fishes Mol Ecol 2009 18 2 169 185 18992003 10.1111/j.1365-294X.2008.03981.x

[B14] Poulin R Morand S The diversity of parasites Q Rev Biol 2000 75 3 277 293 11008700 10.1086/393500

[B15] Lively CM Dybdahl MF Parasite adaptation to locally common host genotypes Nature 2000 405 6787 679 681 10864323 10.1038/35015069

[B16] Parker GA Chubb JC Ball MA Roberts GN Evolution of complex life cycles in helminth parasites Nature 2003 425 6957 480 484 14523438 10.1038/nature02012

[B17] Raeymaekers JAM Huyse T Maelfait H Hellemans B Volckaert FAM Community structure, population structure and topographical specialisation of Gyrodactylus (Monogenea) ectoparasites living on sympatric stickleback species Folia Parasitol 2008 55 3 187 196 19202677 10.14411/fp.2008.026

[B18] Decaestecker E Gaba S Raeymaekers JAM Stoks R Van Kerckhoven L Ebert D De Meester L Host-parasite ‘Red Queen’ dynamics archived in pond sediment Nature 2007 450 7171 870 873 18004303 10.1038/nature06291

[B19] Thompson JN The evolution of species interactions Science 1999 284 5423 2116 2118 10381869 10.1126/science.284.5423.2116

[B20] Eizaguirre C Lenz TL Sommerfeld RD Harrod C Kalbe M Milinski M Parasite diversity, patterns of MHC II variation and olfactory based mate choice in diverging three-spined stickleback ecotypes Evol Ecol 2011 25 3 605 622

[B21] Blais J Rico C van Oosterhout C Cable J Turner GF Bernatchez L MHC adaptive divergence between closely related and sympatric african cichlids PLoS One 2007 28 e734 17710134 10.1371/journal.pone.0000734 PMC1939875

[B22] MacColl ADC Parasites may contribute to ‘magic trait’ evolution in the adaptive radiation of three-spined sticklebacks, Gasterosteus aculeatus (Gasterosteiformes: Gasterosteidae) Biol J Linnean Soc 2009 96 2 425 433

[B23] Karvonen A Seehausen O The role of parasitism in adaptive radiations — when might parasites promote and when might they constrain ecological speciation? International Journal of Ecology 2012 2012 Article ID 280169

[B24] Summers K McKeon S Sellars J Keusenkothen M Morris J Gloeckner D Pressley C Price B Snow H Parasitic exploitation as an engine of diversity Biol Rev 2003 78 4 639 675 14700394 10.1017/s146479310300616x

[B25] Maan ME Van Rooijen AMC Van Alphen JJM Seehausen OLE Parasite-mediated sexual selection and species divergence in Lake Victoria cichlid fish Biol J Linnean Soc 2008 94 1 53 60

[B26] Maan ME van der Spoel M Jimenez PQ van Alphen JJM Seehausen O Fitness correlates of male coloration in a Lake Victoria cichlid fish Behav Ecol 2006 17 5 691 699

[B27] Marcogliese DJ Parasites: small players with crucial roles in the ecological theater EcoHealth 2004 1 151 164

[B28] Taylor MI Turner GF Robinson RL Stauffer JR Sexual selection, parasites and bower height skew in a bower-building cichlid fish Anim Behav 1998 56 379 384 9787029 10.1006/anbe.1998.0795

[B29] Eizaguirre C Lenz TL Major histocompatibility complex polymorphism: dynamics and consequences of parasite-mediated local adaptation in fishes J Fish Biol 2010 77 9 2023 2047 21133915 10.1111/j.1095-8649.2010.02819.x

[B30] Møller AP Parasites and sexual selection - current status of the Hamilton and Zuk hypothesis J Evol Biol 1990 3 5–6 319 328

[B31] Eizaguirre C Lenz TL Traulsen A Milinski M Speciation accelerated and stabilized by pleiotropic major histocompatibility complex immunogenes Ecol Lett 2009 12 1 5 12 19087108 10.1111/j.1461-0248.2008.01247.x

[B32] Gavrilets S Fitness Landscapes and the Origin of Species 2004 Princeton, NJ: Princeton University Press

[B33] Ono H Ohuigin C Tichy H Klein J Major-histocompatibility-complex variation in two species of cichlid fishes from Lake Malawi Mol Biol Evol 1993 10 5 1060 1072 8412649 10.1093/oxfordjournals.molbev.a040055

[B34] Gillardin C Vanhove MPM Pariselle A Huyse T Volckaert FAM Ancyrocephalidae (Monogenea) of Lake Tanganyika: II: description of the first Cichlidogyrus spp. parasites from Tropheini fish hosts (Teleostei, Cichlidae) Parasitol Res 2012 110 1 305 313 21710349 10.1007/s00436-011-2490-5

[B35] Vanhove MPM Snoeks J Volckaert FAM Huyse T First description of monogenean parasites in Lake Tanganyika: the cichlid Simochromis diagramma (Teleostei, Cichlidae) harbours a high diversity of Gyrodactylus species (Platyhelminthes, Monogenea) Parasitology 2011 138 3 364 380 20946697 10.1017/S0031182010001356

[B36] Vanhove MPM Volckaert FAM Pariselle A Ancyrocephalidae (Monogenea) of Lake Tanganyika: I: Four new species of Cichlidogyrus from Ophthalmotilapia ventralis (Teleostei: Cichlidae), the first record of this parasite family in the basin Zoologia 2011 28 2 253 263

[B37] Muterezi Bukinga F Vanhove MPM Van Steenberge M Pariselle A Ancyrocephalidae (Monogenea) of Lake Tanganyika: III: Cichlidogyrus infecting the world’s biggest cichlid and the non-endemic tribes Haplochromini, Oreochromini and Tylochromini (Teleostei, Cichlidae) Parasitol Res 2012 111 2049 2061 22983218 10.1007/s00436-012-3052-1

[B38] Streelman JT Danley PD The stages of vertebrate evolutionary radiation Trends Ecol Evol 2003 18 3 126 131

[B39] Poulin R Phylogeny, ecology, and the richness of parasite communitities in vertebrates Ecol Monogr 1995 65 3 283 302

[B40] Choudhury A Dick TA Richness and diversity of helminth communities in tropical freshwater fishes: empirical evidence J Biogeogr 2000 27 4 935 956

[B41] Mwita C Nkwengulila G Determinants of the parasite community of clariid fishes from Lake Victoria, Tanzania J Helminthol 2008 82 1 7 16 18005464 10.1017/S0022149X07839745

[B42] Luque JL Poulin R Linking ecology with parasite diversity in Neotropical fishes J Fish Biol 2008 72 1 189 204

[B43] Hemmingsen W Halvorsen O MacKenzie K The occurrence of some metazoan parasites of Atlantic cod, Gadus morhua L., in relation to age and sex of the host in Balsfjord (70 degrees N), North Norway Polar Biol 2000 23 5 368 372

[B44] Takemoto RM Pavanelli GC Lizama MAP Luque JL Poulin R Host population density as the major determinant of endoparasite species richness in floodplain fishes of the upper Parana River, Brazil J Helminthol 2005 79 1 75 84 15831117 10.1079/joh2004264

[B45] Machado MH Pavanelli GC Takemoto RM Influence of the type of environment and of the hydrological level variation in endoparasitic infrapopulations of Pseudoplatystoma corruscans (Agassiz) and Schizodon borelli (Boulenger) (Osteichthyes) of the high Parana River, Brazil Revista Brasileira de Zoologia 1995 12 4 961 976

[B46] Konings A Tanganyika cichlids in their natural habitat 1998 El Paso, Texas: Cichlid Press

[B47] Schupke P African Cichlids II. Tanganyika I. Tropheus 2003 Rodgau: Aqualog Verlag A.C.S. GmbH

[B48] Meyer A Knowles LL Verheyen E Widespread geographical distribution of mitochondrial haplotypes in rock-dwelling cichlid fishes from Lake Tanganyika Mol Ecol 1996 5 3 341 350 8688956 10.1111/j.1365-294x.1996.tb00325.x

[B49] Sefc KM Baric S Salzburger W Sturmbauer C Species-specific population structure in rock-specialized sympatric cichlid species in Lake Tanganyika, East Africa J Mol Evol 2007 64 1 33 49 17160645 10.1007/s00239-006-0011-4

[B50] Eschmeyer WN Catalog of Fishes electronic version (14 05 2012) 2012 http://research.calacademy.org/research/ichthyology/catalog/fishcatmain.asp

[B51] Snoeks J Rüber L Verheyen E The Tanganyika problem: comments on the taxonomy and distribution patterns of its cichlid fauna 1994 In: Speciation in ancient lakes. Edited by Verlagsdruckerei Ss. Stuttgart 355 372

[B52] Van Steenberge M Vanhove MPM Risasi DM N’Sibula TM Bukinga FM Pariselle A Gillardin C Vreven E Raeymaekers JAM Huyse T A recent inventory of the fishes of the north-western and central western coast of Lake Tanganyika (Democratic Republic Congo) Acta Ichthyol Piscat 2011 41 3 201 214

[B53] Scholz CA Rosendahl BR Low lake stands in Lakes Malawi and Tanganyika, East Africa, delineated with multifold seismic data Science 1988 240 4859 1645 1648 17745221 10.1126/science.240.4859.1645

[B54] Sturmbauer C Meyer A Genetic divergence, speciation and morphological stasis in a lineage of African cichlid fishes Nature 1992 358 6387 578 581 1501712 10.1038/358578a0

[B55] Koblmüller S Salzburger W Obermüller B Eigner E Sturmbauer C Sefc KM Separated by sand, fused by dropping water: habitat barriers and fluctuating water levels steer the evolution of rock-dwelling cichlid populations in Lake Tanganyika Mol Ecol 2011 20 11 2272 2290 21518059 10.1111/j.1365-294X.2011.05088.x

[B56] Egger B Koblmüller S Sturmbauer C Sefc KM Nuclear and mitochondrial data reveal different evolutionary processes in the Lake Tanganyika cichlid genus Tropheus BMC Evol Biol 2007 7 14 17697335 10.1186/1471-2148-7-137 PMC2000897

[B57] Baric S Salzburger W Sturmbauer C Phylogeography and evolution of the Tanganyikan cichlid genus Tropheus based upon mitochondrial DNA sequences J Mol Evol 2003 56 1 54 68 12569423 10.1007/s00239-002-2380-7

[B58] Mattersdorfer K Koblmüller S Sefc KM AFLP genome scans suggest divergent selection on colour patterning in allopatric colour morphs of a cichlid fish Mol Ecol 2012 21 14 3531 3544 22625655 10.1111/j.1365-294X.2012.05634.x

[B59] Salzburger W Niederstätter H Brandstätter A Berger B Parson W Snoeks J Sturmbauer C Colour-assortative mating among populations of Tropheus moorii, a cichlid fish from Lake Tanganyika, East Africa Proc R Soc B-Biol Sci 2006 273 1584 257 266 10.1098/rspb.2005.3321 PMC1560039 16543167

[B60] Raeymaekers JAM Boisjoly M Delaire L Berner D Räsänen K Hendry AP Testing for mating isolation between ecotypes: laboratory experiments with lake, stream and hybrid stickleback J Evol Biol 2010 23 12 2694 2708 20939859 10.1111/j.1420-9101.2010.02133.x

[B61] Coyne JA Orr HA Speciation 2004 Sunderland, Massachusetts: Sinauer Associates

[B62] Egger B Mattersdorfer K Sefc KM Variable discrimination and asymmetric preferences in laboratory tests of reproductive isolation between cichlid colour morphs J Evol Biol 2010 23 2 433 439 20002244 10.1111/j.1420-9101.2009.01906.x

[B63] Egger B Sefc KM Makasa L Sturmbauer C Salzburger W Introgressive hybridization between color morphs in a population of cichlid fishes twelve years after human-induced secondary admixis J Hered 2012 10.1093/jhered/ess013 22563125

[B64] Egger B Obermüller B Eigner E Sturmbauer C Sefc KM Assortative mating preferences between colour morphs of the endemic Lake Tanganyika cichlid genus Tropheus Hydrobiologia 2008 615 37 48

[B65] Amorim MCP Knight ME Stratoudakis Y Turner GF Differences in sounds made by courting males of three closely related Lake Malawi cichlid species J Fish Biol 2004 65 5 1358 1371

[B66] Blais J Plenderleith M Rico C Taylor MI Seehausen O van Oosterhout C Turner GF Assortative mating among Lake Malawi cichlid fish populations is not simply predictable from male nuptial colour BMC Evol Biol 2009 9 53 19265521 10.1186/1471-2148-9-53 PMC2667177

[B67] Steinwender B Koblmüller S Sefc KM Concordant female mate preferences in the cichlid fish Tropheus moorii Hydrobiologia 2012 682 1 121 130 10.1007/s10750-011-0766-5 PMC3841713 24293682

[B68] Wolinska J Keller B Bittner K Lass S Spaak P Do parasites lower Daphnia hybrid fitness? Limnol Oceanogr 2004 49 4 1401 1407

[B69] Sage RD Heyneman D Lim KC Wilson AC Wormy mice in a hybrid zone Nature 1986 324 6092 60 63 12356091 10.1038/324060a0

[B70] Parris MJ Hybrid response to pathogen infection in interspecific crosses between two amphibian species (Anura: Ranidae) Evol Ecol Res 2004 6 3 457 471

[B71] Lebrun N Renaud F Berrebi P Lambert A Hybrid zones and host-parasite relationships - effect on the evolution of parasitic specificity Evolution 1992 46 1 56 61 10.1111/j.1558-5646.1992.tb01984.x 28564960

[B72] Moulia C Lebrun N Loubes C Marin R Renaud F Hybrid vigor against parasites in interspecific crosses between two mice species Heredity 1995 74 48 52 7852098 10.1038/hdy.1995.6

[B73] Baird SJE Ribas A Macholan M Albrecht T Pialek J de Bellocq JG Where are the wormy mice? A reexamination of hybrid parasitism in the European house mouse hybrid zone Evolution 2012 66 9 2757 2772 22946801 10.1111/j.1558-5646.2012.01633.x

[B74] Malmberg G On the occurrence of Gyrodactylus on Swedish fishes Skrifter utgivna av Södra Sveriges Fiskeriföreningen 1957 1956 19 76

[B75] Taylor MI Meardon F Turner GF Seehausen O Mrosso HDJ Rico C Characterization of tetranucleotide microsatellite loci in a Lake Victorian haplochromine cichlid fish: a Pundamilia pundamilia x Pundamilia nyererei hybdrid Molecular Ecology notes 2002 2 443 445

[B76] Van Oppen MHJ Rico C Deutsch JC Turner GF Hewitt GM Isolation and characterization of microsatellite loci in the cichlid fish Pseudotropheus zebra Mol Ecol 1997 6 387 388 9131814 10.1046/j.1365-294x.1997.00188.x

[B77] Zardoya R Vollmer D Craddock C Streelman T Karl S Meyer A Evolutionary conservation of microsatellite flanking regoins and the phylogeny of cichlid fishes (Pisces: Perciformes) Proc R Soc B-Biol Sci 1996 263 1589 1598 10.1098/rspb.1996.0233 8952095

[B78] Lee WJ Kocher TD Microsatellite DNA markers for genetic mapping in Oreochromis niloticus J Fish Biol 1996 49 169 171

[B79] Parker A Kornfield I Polygynandry in Pseudotropheus zebra, a cichlid fish from Lake Malawi Environ Biol Fishes 1996 47 345 352

[B80] Legendre P Gallagher ED Ecologically meaningful transformations for ordination of species data Oecologia 2001 129 271 280 10.1007/s004420100716 28547606

[B81] Jost L GST and its relatives do not measure differentiation Mol Ecol 2008 17 18 4015 4026 19238703 10.1111/j.1365-294x.2008.03887.x

[B82] Gerlach G Jueterbock A Kraemer P Deppermann J Harmand P Calculations of population differentiation based on GST and D: forget GST but not all of statistics! Mol Ecol 2010 19 18 3845 3852 20735737 10.1111/j.1365-294X.2010.04784.x

[B83] Pritchard JK Stephens M Donnelly P Inference of population structure using multilocus genotype data Genetics 2000 155 2 945 959 10835412 10.1093/genetics/155.2.945 PMC1461096

[B84] Rózsa L Reiczigel J Majoros G Quantifying parasites in samples of hosts J Parasitol 2000 86 2 228 232 10780537 10.1645/0022-3395(2000)086[0228:QPISOH]2.0.CO;2

[B85] Oksanen J Kindt R Legendre P O’Hara RB Stevens MHH 2007 Vegan: Community Ecology Package. R package version 1.8-8 http://r-forge.r-project.org/projects/vegan

[B86] Anderson MJ A new method for non-parametric multivariate analysis of variance Austral Ecol 2001 26 1 32 46

[B87] Coulter GW Coulter GW Composition of the flora and fauna Lake Tanganyika and its Life 1991 Oxford, UK: Oxford University Press 200 274

[B88] Hett ML Report on the Linguatulidae. Zoological Results of the 3rd Tanganyika Expedition (1904–1905) Proceedings of the Zoological Society of London 1924 11 161

[B89] de Beauchamp PM Sur quelques parasites provenant du Congo belge Revue de zoologie et de botanique africaines 1914 4 109 116

[B90] Fryer G The parasitic Crustacea of African freshwater fishes: their biology and distribution J Zool 1968 156 45 95

[B91] Fuhrmann O Baer JG Report on the Cestoda. Zoological results of the Third Tanganyika Expedition (1904–1905) Proceedings of the Zoological Society of London 1925 1 6–7 79 100

[B92] Moore JP Additions to our knowledge of African leeches (Hirudinea) Proceedings of the Academy of Natural Sciences 1938 90 297 360

[B93] Sciacchitano I Contributo alla conoscenza dell’Africa Centrale Revue de zoologie et de botanique africaines 1962 65 3–4 276 381

[B94] Fain A Les Pentastomides de l’Afrique Centrale Annales du Musée Royal de l’Afrique Centrale 1961 8 92 1 115

[B95] Prudhoe S Trematoda, Cestoda and Acanthocephala. Exploration Hydrobiologique du Lac Tanganyika (1946–1947) Résultats Scientifiques 1951 3 2 2 9

